# Replication of a hepatitis C virus replicon clone in mouse cells

**DOI:** 10.1186/1743-422X-3-89

**Published:** 2006-10-28

**Authors:** Susan L Uprichard, Josan Chung, Francis V Chisari, Takaji Wakita

**Affiliations:** 1 The Scripps Research Institute, Department of Molecular and Experimental Medicine, La Jolla, CA 92037 USA; 2 The University of Illinois at Chicago, Department of Medicine and Department of Microbiology and Immunology, Chicago, IL 60612, USA; 3 Department of Microbiology, Tokyo Metropolitan Institute for Neuroscience, Tokyo 183-8526, Japan

## Abstract

**Background:**

Hepatitis C Virus (HCV) is a significant public health burden and small animal models are needed to study the pathology and immunobiology of the virus. In effort to develop experimental HCV mouse models, we screened a panel of HCV replicons to identify clones capable of replicating in mouse hepatocytes.

**Results:**

We report the establishment of stable HCV replication in mouse hepatocyte and fibroblast cell lines using replicons derived from the JFH-1 genotype 2a consensus sequence. Viral RNA replication efficiency in mouse cells was comparable to that observed in human Huh-7 replicon cells, with negative-strand HCV RNA and the viral NS5A protein being readily detected by Northern and Western Blot analysis, respectively. Although HCV replication was established in the absence of adaptive mutations that might otherwise compromise the *in vitro *infectivity of the JFH-1 clone, no infectious virus was detected when the culture medium from full length HCV RNA replicating mouse cells was titrated on Huh-7 cells, suggesting that the mouse cells were unable to support production of infectious progeny viral particles. Consistent with an additional block in viral entry, infectious JFH-1 particles produced in Huh-7 cells were not able to establish detectable HCV RNA replication in naïve mouse cells.

**Conclusion:**

Thus, this report expands the repertoire of HCV replication systems and possibly represents a step toward developing mouse models of HCV replication, but it also highlights that other species restrictions might continue to make the development of a purely murine HCV infectious model challenging.

## Background

Hepatitis C virus (HCV) is an enveloped, positive-strand RNA virus that causes acute and chronic hepatitis [1]. Between 70–90% of those who become infected fail to clear the virus and remain chronically infected with the risk of developing liver cirrhosis and hepatocellular carcinoma [2]. Unfortunately, there is no vaccine available to prevent this infection, and the only approved treatment has toxic side effects and is only effective in a subset of patients [3,4].

Even though recent work has led to the development of *in vitro *HCV infection systems, which allow for molecular analysis of the entire viral life cycle [5-7], the study of the immunobiology and pathogenesis of HCV still requires the development of genetically defined small animal models. One obstacle to the development of HCV mouse models has been the limited host range of the virus. The restrictions that block HCV infection in mice are not well defined, but appear to involve multiple steps such as viral entry and genome replication.

**Figure 1:**
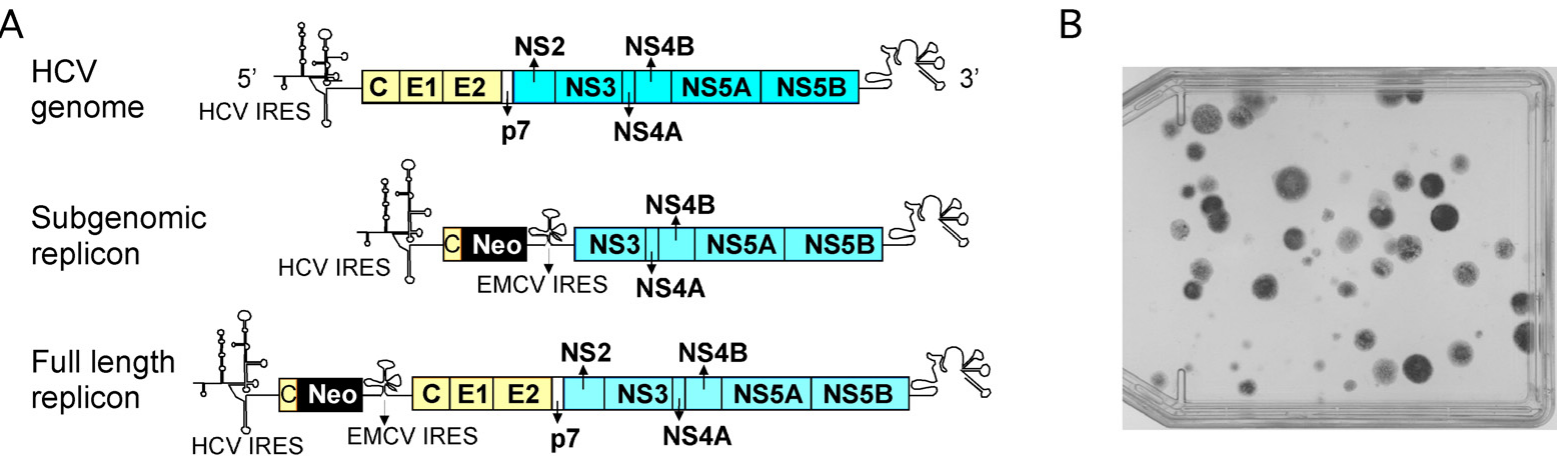
(A) Schematic diagram of HCV genomic and replicon RNA. (B) Representative crystal violet staining of G418-resistant colony formation in MMHD3 mouse hepatocytes after transfection with sgJFH-1 HCV RNA. Notably, although MMH cells exhibit relatively low transfection efficiency, numerous G418-resistant colonies form following transfection of HCV replicon RNA.

Notably however, HCV replicons based on engineered viral genomes into which the antibiotic resistant marker neomycin phosphotransferase (neo) has been inserted [8](Fig. 1A) provide a means of experimentally by-passing viral entry and actively selecting for HCV replication after transfection of RNA into cells. The ability to select for cells replicating the neo-expressing replicon RNA led to the discovery that efficient replication of most HCV replicons in cell culture requires adaptive mutations in the viral genome [9-13]. Although HCV replication initially could only be achieved in the human hepatoma cell line, Huh-7, the ability to select for replication enhancing mutations eventually led to the establishment of HCV replication in other hepatic (HepG2 and IMY-N9 [14]) and nonhepatic (HeLa [15,16] and HEK293 [15,17]) human cell lines.

Unlike other published studies that focus exclusively on HCV replication in human and/or primate cell lines, Zhu et al (2003) further demonstrated that replication of the HCV-N genotype 1b subgenomic replicon could be initiated in one of the several mouse cell lines tested. However, this replication could only be established in a single mouse cell line after transfection of total RNA extracted from HeLa cells that were already replicating the adapted replicon (i.e. total human cellular RNA presumably containing a quasispecies of HCV replicons)[16]. In contrast, HCV replication could not be initiated in these mouse cells by transfection of *in vitro *transcribed replicon RNA generated from either the parental replicon construct or from any of the "adapted" replicon clones isolated from their original mouse replicon cells. Hence, no "mouse-permissive" HCV replicon clone was identified.

Because the development of HCV mouse models would be greatly facilitated by the identification of defined HCV clone(s) capable of establishing and maintaining replication in mice, we assembled a panel of HCV replicons derived from different HCV genotypes and assessed their ability to replicate in mouse cells. We show that JFH-1 genotype 2a subgenomic and full length replicons are able to stably replicate in multiple mouse hepatocyte and fibroblast cell lines following transfection of the replicon RNA. Although the HCV replication achieved in mouse hepatocytes was not dependent on adaptive mutations that might compromise the infectivity of the viral clone, no infectious HCV was detected in the media of mouse cells replicating full length HCV RNAs, nor were infectious JFH-1 virus particles produced in Huh-7 cells able to infect naïve mouse cells. Thus, these mouse cells are permissive for JFH-1 HCV replication, but exhibit blocks in both viral entry and production of infectious particles.

## Results

### Establishment of G418-resistant mouse hepatocyte colonies after transfection of subgenomic replicons encoding the neomycin selection gene

Because different HCV replicon clones exhibit a range of replication efficiencies in Huh-7 cells, we hypothesized that different replicons might also display differences in their ability to replicate in mouse cells. Thus, we screened a panel of subgenomic HCV replicons derived from genotypes 1b, 1a, and 2a for their ability to confer G418 resistance to mouse hepatocytes *in vitro*. We initially screened for HCV replication in immortalized Met Mouse Hepatocytes (MMH) cells; however, because HCV replication efficiency within different Huh-7 cell lines can vary, we tested HCV replication in two independently derived MMH cell lines, MMHD3 [18] and MMH1-1 [19].

Replicon RNA from the clones listed in Table 1 was transcribed *in vitro *and electroporated into MMH cells. Transfected cells were then treated with 500 μg/ml G418, which was the minimal concentration required to effectively kill untransfected control cell cultures. Despite the fact that G418-resistent Huh-7 cell colonies survived the selection process after transfection with all of the replicon RNAs tested, RNA transcribed from the HCV genotype 1b clones (Con1 and HCV-N) and the HCV genotype 1a H77 clone did not generate G418-resistent mouse cell colonies (data not shown). In contrast, transfection of genotype 2a subgenomic (sg) JFH-1 RNA produce G418-resistant mouse hepatocyte colonies in both MMH cell lines in multiple independent transfection experiments (Fig. 1B).

**Table 1:** HCV Replicons Screened

Genotype	Clone
1b	sgCon1 WT
1b	sgCon1 S1179I
1b	sgHCV-N
1a	sgH77
2a	sgJFH-1

sg = subgenomic

### Expression and replication of sgJFH-1 HCV replicons in immortalized mouse hepatocytes

**Figure 2:**
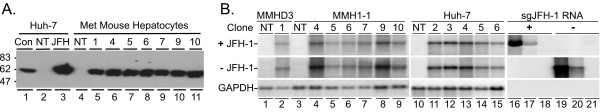
HCV protein and RNA detection in immortalized mouse hepatocytes. (A) Western Blot detection of HCV NS5A. Cell lysate was harvested from individual cell clones and resolved by SDS-PAGE. Samples include cell lysate from Huh-7 sgCon1 (lane 1); non-transfeted (NT) Huh-7 (lane 2); Huh-7 sgJFH-#2 (lane 3); NT MMHD3 cells (lane 4); MMHD3 sgJFH#1 (lane 5); and MMH1-1sgJFH#4, 5, 6, 7, 9, and 10 (lanes 6–11). (B) Northern Blot detection of sgJFH-1 RNA. Total RNA was isolated from non-transfected (NT) and individual MMHD3 (lanes 2), MMH1-1 (lanes 4–9), and Huh-7 (lanes 11–15) sgJFH-1 replicon clones. Positive-strand (top panel) and negative-strand (middle panel) HCV RNA was detected with ^32^P-labeled strand-specific riboprobes. Cellular GAPDH was detected with a ^32^P-labeled cDNA probe (bottom panel). Serial dilutions of *in vitro *transcribed positive-strand and negative-strand sgJFH-1 RNA (10^9^, 10^8^, 10^7^) are shown in lanes 16–18 and lanes 19–21, respectively.

To confirm the expression and replication of HCV in MMHD3 and MMH1-1 cells, G418-resistant cell colonies were expanded for further analysis. Western Blot analysis of total cell lysate verified that the viral NS5A protein was present in each of the MMHD3 and MMH1-1 replicon cell clones (Fig. 2A, lanes 5, and 6–11, respectively). Yet, constitutive HCV protein expression was not due the integration of an HCV transgene, as PCR analysis on cellular genomic DNA revealed no evidence of JFH-1 sequences (data not shown).

Clones were also examined for the presence of HCV RNA by strand-specific Northern Blot. Positive-strand (Fig. 2B, top panel) and negative-strand (Fig. 2B, middle panel) HCV RNA was detected not only in Huh-7 control cell clones (Fig. 2B, lanes 11–15), but also in the mouse clones derived from MMHD3 and MMH1-1 cells (Fig. 2B, lanes 2 and 4–9, respectively). Although the signal intensity observed with these independently labeled and hybridized riboprobes can not be compared to determine the ratio of positive-strand to negative-strand HCV RNA, both probes demonstrated stringent strand-specific hybridization when incubated with membranes containing serial dilutions (10^9^, 10^8^, and 10^7 ^copies) of *in vitro *transcribed positive-strand (Fig. 2B, lanes 16–18) and negative-strand (Fig. 2B, lanes 19–21) sgJFH-1 RNA controls, clearly demonstrating that negative-strand HCV RNA replication intermediates were being produced in the clones.

RT-QPCR analysis of the same RNA samples indicated that HCV RNA levels among the mouse sgJFH-1 replicon clones ranged from 2.9 × 10^6 ^– 7.0 × 10^7 ^copies per microgram (copies/μg) of total cellular RNA. This was similar to the 7.4 × 10^6 ^– 8.5 × 10^7 ^copies/μg observed in the human sgJFH-1 replicon clones. Thus, the average 2.0 × 10^7 ^HCV RNA copies/μg calculated in the mouse cells was comparable to the average 5.0 × 10^7 ^HCV RNA copies/μg detected in the Huh-7 clones indicating that while the level of the HCV RNA does vary between individual cells clones, no statistical difference in RNA level was detectable between mouse and human cells.

### Adaptive mutations are not required for replication of sgJFH-1 replicons in mouse hepatocytes

**Figure 3:**
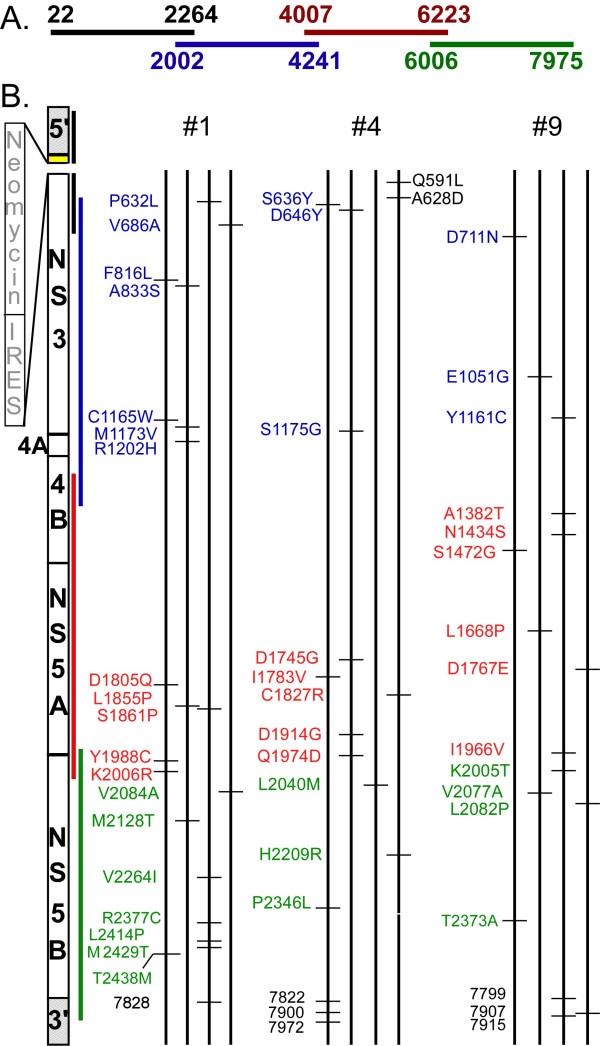
Sequence analysis of sgJFH-1 replicons in mouse hepatocytes. (A) PCR primer sets used to amplify overlapping HCV DNA segments. (B) Twelve clones of each fragment were sequenced with an ABI automatic DNA sequencer. Mutations within viral proteins are indicated by the amino acid position of the mutation within the context of the full length HCV open reading frame. Mutations in the 3' UTR are designated by their nucleotide position in the context of the full length HCV genome.

Because adaptive mutations are required for efficient replication of many HCV replicons in cell culture [9-11,20-22], we sequenced replicon clones obtained from our mouse cell lines to determine whether the population of sgJFH-1 replicons present in the MMH cells had acquired any adaptive mutations. RNA was extracted from 3 of the MMH replicon cell lines (MMHD3-sgJFH#1 and MMH1-1sgJFH#4 and #9). After reverse transcription, 4 PCR primer sets were used to amplify overlapping segments spanning the sgJFH-1 cDNA (Fig. 3A). These PCR products were ligated into the pGEMT-Easy vector and 12 individual clones of each fragment were sequenced. Although random mutations were observed throughout the cloned genomes, none of the changes were detected in more than 1 of the 12 clones sequenced (Fig. 3B), supporting the conclusion that adaptive mutations had not become established in the mouse replicon population. Also consistent with the absence of replicon adaptive mutations, total RNA isolated from both MMH and Huh-7 replicon cells containing comparable copies of sgJFH RNA (as determined by RT-QPCR) formed equivalent numbers of G418-resistant colonies/μg of viral RNA when re-transfected into naïve MMH cells (data not shown).

To determine if the initial establishment of the mouse replicon clones had selected for adapted MMH cells that were more permissive for HCV replication, we also "cured" the sgJFH-1 replicon from our MMH replicon cells with a 3 week treatment of IFNα (1000 U/ml) and IFNγ (500 U/ml), but subsequent sgJFH-1 RNA transfections into these "cured" (e.g. G418-sensitive, HCV-negative) mouse cells did not produce higher numbers of G418-resistent colonies/μg of input RNA compared to parallel transfections into the parental MMH cell lines indicating that we had not selected MMH cells that more efficiently allow for establishment of HCV replication (data not shown).

### Replication of sgJFH-1 HCV in other mouse cell lines

**Figure 4:**
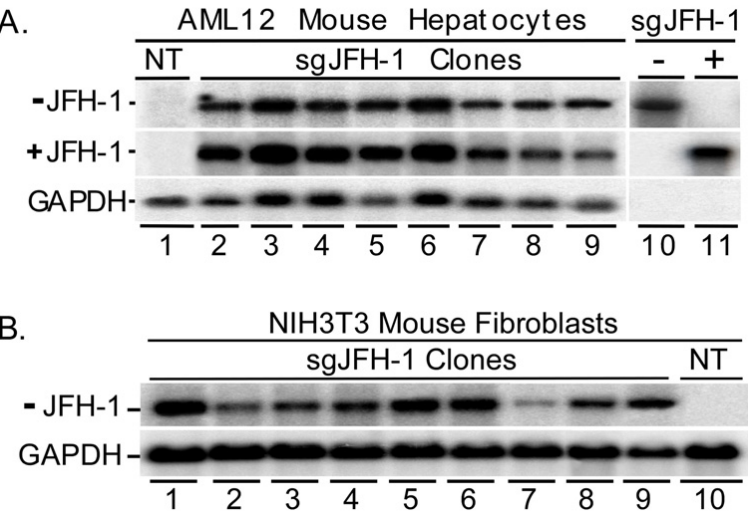
Northern Blot detection of sgJFH-1 RNA in AML12 mouse hepatocytes and NIH3T3 mouse fibroblasts. (A) Negative-strand HCV (top panel), positive-strand HCV (middle panel), and cellular GAPDH (bottom panel) was detected by Northern Blot analysis of total RNA isolated from non-transfected (NT) parental AML12 cells (lane 1) and 8 individual sgJFH-1 RNA transfected clones (lanes 2–9). *In vitro *transcribed positive-strand (lane 10) and negative-strand (lane 11) sgJFH-1 RNA was included as controls. (B) Northern Blot analysis for negative-strand HCV (top panel) and cellular GAPDH (bottom panel) was performed on total RNA isolated from non-transfected (NT) parental NIH3T3 cells (lane 10) and 9 randomly chosen sgJFH-1 RNA transfected clones (lanes 1–9).

Because the sgJFH-1 replicon was able to replicate in MMH cells without any apparent viral or cellular adaptive mutations, we proceeded to determine if this ability was restricted to MMH cells or if the JFH-1 clone could replicate in other mouse cell lines. Hence, we transfected *in vitro *transcribed sgJFH-1 RNA into mouse AML12 hepatocytes as well as mouse NIH3T3 embryonic fibroblasts and selected for cells that were able to support replication of the neo-expressing replicon. Figure 4A shows strand-specific HCV Northern Blot analysis that verifies the presence of both positive-strand (Fig. 4A, top panel) and negative-strand (Fig. 4A, middle panel) HCV RNA in all the AML12 mouse hepatocyte cell clones. Similarly, negative strand-specific HCV Northern Blot analysis of total RNA from 9 NIH3T3 replicon cell clones also confirmed the presence of negative-strand HCV RNA in these non-hepatic mouse cells demonstrating that the sgJFH-1 HCV clone can replicate in a variety of mouse cell lines (Fig. 4B).

### Replication of full length JFH-1 HCV replicons in mouse cells

**Figure 5:**
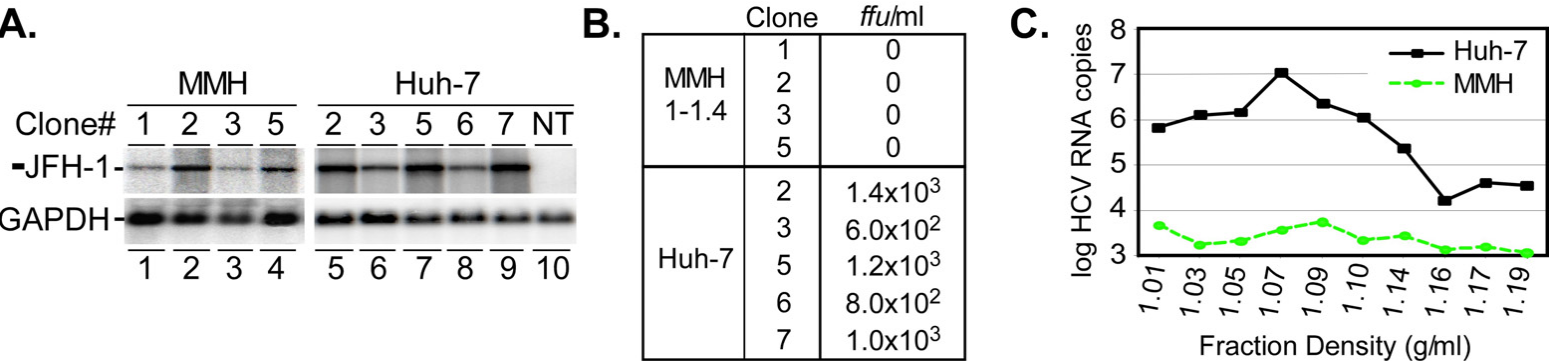
Intracellular and extracellular HCV RNA produced by flJFH-1 mouse replicon clones. (A) Northern Blot analysis for negative-strand HCV (top panel) and cellular GAPDH (bottom panel) was performed on total RNA isolated from MMH1-1 replicon clones (lanes 1–4), Huh-7 replicon clones (lanes 5–9), and non-transfected (NT) Huh-7 cells (lane 10). (B) HCV infectivity titer in the culture medium of the same MMH and Huh-7 clones was determined by incubating serial dilutions on naïve Huh-7 cells and performing immunofluorescence for HCV 3 days post-inoculation, as previously described [5]. The titer is expressed as NS5A-positive focus-forming units (*ffu*) per ml of medium. (C) RT-QPCR detection of extracellular HCV RNA following sucrose density gradient centrifugation.

To determine if full length HCV replication also could be established in mouse cells, *in vitro *transcribed full length (fl) JFH-1 replicon RNA was electroporated into MMH1-1 and Huh-7 cells, and cells supporting replicon replication were selected with 500 μg/ml G418. Northern Blot analysis of total RNA isolated from the resulting G418-resistant clones confirmed that negative-strand flJFH-1 RNA was present in both MMH1-1 (Fig. 5, lanes 1–4) and Huh-7 (Fig. 5, lanes 5–10) transfected cultures. However, unlike sgJFH replicon clones in which HCV RNA levels were equivalent between human and mouse cells clones, RT-QPCR analysis indicated that HCV RNA levels were approximately 3-fold lower in flJFH-1 MMH1-1 cell clones (9.5 × 10^4 ^– 7 × 10^5 ^copies/μg total RNA) compared to flJFH-1 Huh7 cell clones (3.1 × 10^5 ^– 1.9 × 10^6 ^copies/μg total RNA).

### Lack of infectious HCV particle entry into and secretion from mouse hepatocytes

Because the flJFH-1 is capable of producing infectious HCV particles, we proceeded to determine whether the MMH1-1 mouse cells were able to secrete infectious HCV particles and/or were permissive for flJFH-1 entry. To determine if the flJFH-1 replicon clones were secreting infectious HCV particles that could transmit G418-resistance, the culture media from individual clones was collected and used to inoculate naive Huh-7 and MMH1-1 cells. Although G418-resistant colonies formed when Huh-7 cells were inoculated with the supernatant from Huh-7 flJFH-1 replicon cells, the culture supernatant from MMH1-1 flJFH-1 replicon cells did not confer G418-resistance to Huh-7 cells suggesting that the mouse cells were not able to secrete infectious HCV particles. Titration of the same media on Huh-7 cells further confirmed that all the Huh-7 flJFH-1 cell clones were secreting infectious HCV particles (expressed as foci forming units per ml), while no infectious HCV was detected in the media from MMH1-1 flJFH-1 cells, even after 50-fold concentration by centrifugal filtration (Fig. 5B).

Notably, inoculation of naïve MMH1-1 cells with media from either Huh-7 flJFH-1 or MMH1-1 flJFH-1 cell cultures did not confer G418-resistence to the mouse cells confirming that HCV particles are unable to enter mouse cells. Importantly, inoculation with high titer authentic HCV virus produced in Huh-7 cells also failed to establish JFH-1 replication in MMH1-1, AML12, or NIH3T3 mouse cells (data not shown).

To determine whether the lack of detectable infectivity in the culture medium of flJFH-1 MMH1-1 replicon clones was due to an inability to secrete HCV particles or due to the secretion of non-infectious HCV particles, the culture supernatant from Huh-flJFH#7 and MMH1-1-flJFH#2 cultures were subjected to sucrose density gradient centrifugation. Fractions were collected, total RNA extracted, and RT-QPCR analysis performed to determine the amount of viral RNA in each fraction (Fig. 5C). HCV RNA in the supernatant of the Huh-flJFH#7 cells was detected over a broad density range of 1–1.15 g/ml, with a predominant peak at 1.07 g/ml, supporting the conclusion that the viral RNA was associated with enveloped particles (Fig. 5C). This is similar to the HCV RNA density profile reported for *in vitro*-derived infectious JFH-1 particles from which RNA was observed in density fractions from 1–1.15 g/ml with a peak at 1.09–1.10 g/ml [5,6]. In contrast, there was no specific low density peak of HCV RNA in the media of MMH1-1flJFH#2 cells and total viral RNA levels in the supernatant were significantly lower than that seen the in Huh-7 replicon cell supernatant. Hence, these results support the conclusion that mouse hepatocytes do not efficiently secrete enveloped, flJFH-1 RNA-containing particles.

## Discussion

In this study, we have shown that replicon clones derived from the HCV JFH-1 consensus cDNA can establish and maintain efficient replication in both mouse hepatocytes (e.g. MMHD3, MMH1-1, AML12) and mouse embryonic fibroblasts (e.g. NIH3T3). This discovery reveals a previous unrealized breadth in the host range of the JFH-1 HCV clone, and expands our ability to develop experimental HCV model systems. In addition to observing stable expression of viral proteins and replication of viral RNA (Fig. 2), we found that HCV replication in mouse hepatocytes is not dependent on adaptive mutations in the viral genome (Fig. 3), but is sensitive to inhibition by interferons (data not shown) and the HCV-specific small molecule inhibitor, BILN2061 (data not shown). Taken together, these results demonstrate replication of non-adapted HCV replicon clones in mouse cells, and they potentially provide the basis for the production of a mouse model of HCV replication.

While Zhu et al., (2003) previously provided evidence that HCV replication in mouse hepatocytes was possible, the data presented here differs from that report in three fundamental ways. First, Zhu et al., (2003) could only initiate HCV replication in mouse cells by transfection with a HCV RNA quasispecies obtained from previously established HeLa replicon cells, but they were not able to establish replication in mouse cells with any individual replicon clone. In contrast, we have been able to identify a defined HCV replicon clone capable of replicating in various mouse cell lines. The identification of a specific mouse-permissive HCV clone is advantageous because it facilitates mouse model development and allows for reverse-genetics experimentation. Second, whereas replication of the subgenomic 1b HCV-N replicon reported by Zhu et al., (2003) was restricted to one specific mouse cell line, we have been able to establish JFH-1 HCV replication in multiple mouse cell lines significantly enhancing the likelihood that future *in vitro *and *in vivo *experimental HCV mouse models based on the JFH-1 clone might be possible. Finally, while the previous success was dependent on cell culture adaptive mutations that prevent HCV infectivity [23], we have demonstrated that replicons derived from the JFH-1 consensus sequence can replicate in mouse hepatocytes, in the absence of adaptive mutations that might otherwise alter the infectivity of the clone.

The ability of the JFH-1 clone to replicate efficiently in tissue culture in the absence of adaptive mutations has recently led to the development of *in vitro *HCV infection models in Huh-7 cells [5-7] and the demonstration that full length HCV replicons are capable of producing infectious viral particles (Fig. 5B–C). Likewise it provided us the opportunity to study the ability of mouse cells that support JFH-1 replication to uptake and secrete infectious HCV JFH-1 particles. Consistent with previous HCV pseudoparticles data, we observed no evidence that HCV particles were able to productively enter mouse cells, even when we used G418 treatment to select for cells that might have become HCV positive following inoculation. More unexpectedly, we also observed that mouse hepatocytes replicating infectious JFH-1 RNA were not able to secrete infectious HCV RNA containing particles. Though it is possible there were low levels of infectious particles present that were below the detection limits of our assays, sucrose density gradient analysis and RT-QPCR analysis of HCV RNA isolated from the mouse culture supernatants supports the conclusion that very few, if any, enveloped, HCV RNA-containing particles were being secreted from these mouse cells (Fig. 5C). This may reflect an inability to assemble infectious particles, secrete viral particles, or both. Hence, in addition to productive viral entry, infectious particle production appears to be another aspect of the HCV life cycle that may not be readily recapitulated in mouse hepatocytes. Clearly, elucidating the basis of this phenomenon may help us understand the key steps required for infectious HCV particle secretion in human cells.

## Conclusion

The data presented in this report demonstrate that JFH-1 derived HCV replicon clones can efficiently replicate in mouse cells. While this discovery certainly represents an advance in our ability to develop mouse models of HCV replication, further analysis of infectious JFH-1 HCV entry into and egress from mouse cells also identified at least 2 other aspects of the HCV life cycle that are not supported by the mouse hepatocytes tested. Such species restrictions will likely continue to make the development of a purely murine HCV infectious model challenging.

## Methods

### HCV constructs

Constructs containing the non-adapted wild type (pHCVrep1b BartMan_AvaII) and Huh-7 cell adapted [pHCVrep1bBB7 (S1179I)] HCV Con1 genotype 1b subgenomic replicons [9] and the genotype 1a H77 subgenomic replicon [H/SG-Neo(L+I)] [22] were provided by Dr. Charles Rice (Rockefeller University, NY). The pNNeo/3-5B(SI) construct encoding the genotype 1b HCV-N subgenomic replicon was provided by Dr. Stanley Lemon (University of Texas Medical Branch, Galveston)[13]. The constructs pSGR-JFH1, pFGR-JFH1, and pJFH-1 containing the sgJFH-1 HCV genotype 2a JFH-1 replicon, the full length (fl)JFH-1 replicon, and the genomic JFH-1 viral clone have been described [7,24,25]. In all constructs, the HCV cDNA is located at the 1+ position 5' of the T7 promoter. Plasmids were linearized at the 3' end of the HCV cDNA and used as a template for *in vitro *transcription by T7 RNA polymerase (MEGAscript; Ambion, Austin, TX). To generate strand-specific RNA probes, a 1 kb fragment of the JFH-1 NS5B coding region (HindIII-EcoRV) was cloned into the pBSKII+ vector to allow for T7 and SP6-driven transcription of JFH-1 negative and positive strand probes, respectively.

### Cell culture

Huh-7 human hepatocytes and NIH3T3 mouse fibroblasts (CRL-1658; ATCC, Manassas, VA) were maintained in DMEM supplemented with 10% fetal calf serum and Penicillin-Streptomycin-Glutamine (100×, liquid)(Gibco Invitrogen Corporation, Carlsbad, CA). AML12 mouse hepatocytes (ATCC; CRL-2254) were maintained in DMEM/F12 supplemented with 10% fetal calf serum, Penicillin-Streptomycin-Glutamine (100×, liquid), insulin-transferrin-selenium (100×, liquid)(Gibco Invitrogen), and 40 ng/ml dexamethasone (Sigma, St. Louis, MO). MMHD3 (Met Murine Hepatocyte) cells were obtained from Marco Tripodi (Università La Sapienza, Italy)[18]. This immortalized mouse hepatocyte cell line was derived from the liver of transgenic mice, which express the constitutively active cytoplasmic domain of the human hepatocyte growth factor receptor (cMet) in their livers. The MMH1-1 cells were independently derived from double transgenic cMet transgenic mice that also have a hepatitis B virus (HBV) transgene [19]. Although these cells contain a HBV transgene, expression and replication of HBV in these cells only occurs after the cells become confluent and are further differentiated in the presence of 2% DMSO for 8 days [19]. All of the experiments presented here were performed on subconfluent cell cultures in the absence of DMSO; therefore, no HBV replication was occurring in these cells (data not shown). Both MMHD3 and MMH1-1, were plated on collagen I Biocoat dishes (Becton Dickinson, Franklin Lakes, NJ) in RPMI 1640 (Gibco Invitrogen) supplemented with 10% fetal calf serum (Gibco Invitrogen), 55 ng/ml EGF (Becton Dickinson), 16 ng/ml IGF-II (Calbiochem, San Diego, CA), 10 μg/ml insulin (Sigma) and Penicillin-Streptomycin-Glutamine (100×, liquid)(Gibco Invitrogen)[19]. G418 was added to culture media as indicated (Invitrogen).

### HCV RNA transfection and G418-resistant colony formation

*In vitro *transcribed HCV RNA or total cell RNA was transfected into cells using a modified electroporation protocol [10]. Trypsinized cells were washed twice with serum-free medium and resuspended to a final concentration of 1 × 10^7 ^cells/ml. One to ten micrograms of HCV RNA was then mixed with 0.4 ml of the cells in a 4 mm cuvette. A Gene Pulser system (BioRad Laboratories, Hercules, CA) was used to deliver the following single pulses: Huh-7 (0.27 kV, 100 OHMS, 960 μF); Met-based and AML12 cells (0.45 kV, 100 OHMS, 960 μF); NIH3T3 (0.27 kV, 100 OHMS, 960 μF). For the generation of replicon clones, transfected cultures were maintained in the presence of G418 at a concentration of 500 μg/ml until all cells died or distinct G418-resistant cell colonies formed. To visualize colony formation, cells were fixed and stained with crystal violet.

### RNA analysis

Total cell RNA was isolated by the guanidine thiocyanate method using standard protocols [26]. RNA was resolved in 1% agarose, 2.2 M formaldehyde gels and transferred to nylon membrane (Schleicher & Schuell, Keene, NH). Membranes were cut across the 28S ribosomal band so that the top of the blot could be hybridized with ^32^P-labeled strand-specific riboprobes (MAXIscript; Ambion), while the bottom was hybridized with ^32^P-labeled cellular GAPDH cDNA probe (Random Prime Synthesis; Invitrogen, Carlsbad, CA). Hybridized probe was visualized using a storage phosphor system (Cyclone; Packard Instrument Co.). Alternatively, 1 μg of RNA was DNAse treated (DNA-*free *reagent; Ambion) for reverse transcription quantitative polymerase chain reaction (RT-QPCR). RNA was used for cDNA synthesis using the TaqMan reverse transcription reagents (Applied Biosystems, Foster City, CA), followed by real-time PCR quantification using a BioRad iCycler (BioRad Laboratories). HCV and GAPDH transcript levels were determined relative to a standard curve comprised of serial dilutions of plasmid containing the HCV cDNA or the mouse GAPDH gene. The PCR primers used to detect JFH-1 (GenBank AB047639) were 5'-TCTGCGGAACCGGTGAGTA-3' (sense) and 5'-TCAGGCAGTACCACAAGGC-3' (antisense). The PCR primers used to detect H77C (GenBank AF011751) were 5'-GTCTGCGGAACCGGTGAG-3' (sense) and 5'-GGCATTGAGCGGGTTTATC-3' (antisense). The PCR primers used to detect both genotype 1b HCV clones (Genbank AJ242652 and AF139594) were 5'-ATGGCGTTAGTATGAGTGTC-3' (sense) and 5'-GGCATTGAGCGGGTTGATC-3' (antisense). The PCR primers used to detect mouse GAPDH (GenBank M32599) were 5'-TCTGGAAAGCTGTGGCGTG-3' (sense) and 5'-CCAGTGAGCTTCCCGTTCAG-3' (antisense).

### Western Blot analysis

Cells were harvested in RIPA buffer (50 mM Tris-HCl, pH 7.4, 1% NP-40, 0.25% Na-deoxycholate,150 mM NaCl, 1 mM EDTA) supplemented with a protease inhibitor cocktail (Roche Applied Science, Indianapolis). Fifty micrograms of protein was resolved by SDS-PAGE and transferred to Hybond nitrocellulose membranes (Amersham Pharmacia, Piscataway, NJ). Membranes were sequentially blocked with 5% Nonfat Milk, incubated with a 1:500 dilution of the polyclonal rabbit NS5A antibody, M15 (provided by Dr. Michael Houghton, Chiron, Emeryville, CA), washed 3 times with PBS/0.05% Tween20, incubated with horseradish peroxidase-conjugated goat anti-rabbit antibody (Pierce, Rockford, Illinois), and washed again. Bound antibody complexes were detected with SuperSignal chemiluminescent substrate (Pierce).

### Sequencing HCV replicons

Total cell RNA was extracted from 3 mouse replicon cell lines (MMHD3sgJFH#1, MMH1-1sgJFH#4, and MMH1-1sgJFH#9). Reverse transcription was carried out with random hexamers using 1 μg of RNA (Transcriptor Reverse Transcriptase; RocheApplied Sciences, Indianapolis, IN). HCV DNA was subsequently amplified with high fidelity Tgo polymerase (Roche). Four PCR primer sets were used to amplify overlapping segments spanning positions 22 to 2264, 2002 to 4241, 4007 to 6223, and 6006 to 7975 of the sgJFH-1 replicon (Fig. 3A). The PCR products were cloned into the pGEM-T Easy plasmid (Promega, Madison WI), and 12 clones of each fragment (4 from each cells line) were sequenced with an ABI automatic DNA sequencer using the M13 forward and reverse primers in the vector as well as 20–21 bp sequencing primers located within the HCV subgenomic replicon at nucleotide positions: 1252, 1790, 2053, 2290, 2571, 2820, 3269, 3836, 4256, 4634, 4813, 5215, 5425, 6148, 6555, and 7416.

### Titration of infectious HCV

Cell supernatants were serially diluted in complete medium and used to infect naïve MMH and Huh-7 cells in 96-well plates. The level of HCV infection was determined 3 days post-infection by immunofluorescence staining for HCV NS5A as previously described [5]. The viral titer is expressed as focus-forming units per milliliter of supernatant (*ffu*/ml), determined by the number of NS5A-positive foci detected at the highest HCV-positive dilution.

### Sucrose density gradient analysis

Sucrose density-gradient ultracentrifugation was performed as described [5]. Supernatant from flJFH-1 replicon cultures was collected, centrifuged at 4,000 rpm for 5 min to remove cellular debris, and concentrated by centrifugal filteration when indicated (Amicon Ultra; Millipore, Billerica, MA). Samples were loaded onto TNE buffer (50 mM Tris·HCl, pH 8;100 mM NaCl;1 mM EDTA)-based 20–60% sucrose gradient and centrifuged at 120,000 × *g *for 16 h at 4°C using a SW60 rotor in a Beckman Coulter L8-80 Ultracentrifuge (Fullerton, CA). Fractions were collected from the bottom of the gradient, and analyzed for HCV RNA by RT-QPCR.

## Note

While this manuscript was being prepared, Chang et al., (JVirol. 2006 Aug;80:7364-74) published a report consistent with these data showing that JFH-1 replicons can replicate in mouse embryonic fibroblasts (MEFs).

## Competing interests

The author(s) declare that they have no competing interests.

## Authors' contributions

SLU conceived and designed the study, obtained funding, and then performed the experiments. JC did the sequence analysis of sgJFH-1 from mouse cells and cloned the JFH-1 RNA probe template plasmid. FVC participated in data analysis, revising of the manuscript, and provided funding for JC. TW provided the critical unpublished reagents, participated in data analysis, and revising of the manuscript. All authors read and approved the manuscript.
